# Human type 1 conventional dendritic cells contribute to skin transplant rejection

**DOI:** 10.1016/j.ajt.2025.04.016

**Published:** 2025-04-24

**Authors:** Thiago J. Borges, Catherine A.A. Lee, Kyla Mucciarone, Karina Lima, Isadora T. Lape, Mauricio Lima-Filho, Bruno Ayoama, Branislav Kollar, Rodrigo B. Gassen, Cristina Bonorino, Simon G. Talbot, Bohdan Pomahac, Christine G. Lian, George F. Murphy, Leonardo V. Riella

**Affiliations:** 1 Center for Transplantation Sciences, Department of Surgery, Massachusetts General Hospital, Harvard Medical School, Boston, Massachusetts, USA; 2 Department of Pathology, Brigham and Women’s Hospital, Harvard Medical School, Boston, Massachusetts, USA; 3 Department of Plastic and Hand Surgery, University of Freiburg Medical Center, Medical Faculty of the University of Freiburg, Freiburg, Germany; 4 Immunotherapy Laboratory - (LAIT) - Department of Basic Health Sciences of Federal University of Health Sciences of Porto Alegre, UFCSPA, Porto Alegre, Brazil; 5 Division of Plastic and Reconstructive Surgery, Brigham and Women’s Hospital, Harvard Medical School, Boston, Massachusetts, USA; 6 Division of Plastic and Reconstructive Surgery, Department of Surgery, Yale New Haven Hospital, Yale School of Medicine, New Haven, Connecticut, USA

**Keywords:** vascularized composite allograft, dendritic cells, skin, immune modulation

## Abstract

The skin is the most immunogenic tissue in transplantation and the most difficult tissue in which to induce immune modulation. Batf3-dependent type 1 conventional dendritic cells (cDC1s) are important in initiating rejection in murine skin transplantation. In humans, the CD141^+^ cDC1 subset is the functional counterpart of the murine Batf3-dependent cDC1s. However, their contribution to the rejection of human skin allografts remains unknown. Using samples from human face and upper extremity transplant recipients, we demonstrated that CD141^+^ cDC1s are increased and more activated in human skin grafts than native skin tissue from the same individual. Moreover, circulating and tissue CD141^+^ cDC1s were elevated at rejection time points. Local modulation of graft CD141^+^ cDC1s decreased HLA-DR expression and increased regulatory T cells, which correlated with a decreased presence of skin allogeneic T cells in a humanized transplantation model. Thus, CD141^+^ cDC1s play an important role in rejecting human skin allografts, and their local modulation is a promising therapeutic approach.

## 1. Introduction

The skin is the major component of and the primary tissue target involved in the rejection of vascularized composite allotransplantation (VCA), such as face and extremity transplantation.^1^ The presence of a large number of antigen-presenting cells (APCs) in the skin creates a highly immunogenic microenvironment.^2,3^ Indeed, the skin is the most difficult tissue in which to induce immune modulation in transplantation compared to other organ transplants, such as kidney and heart.

Dendritic cells (DCs) are the major APC subset responsible for initiating adaptive alloimmunity.^4^ After transplantation, inflammation and ischemic tissue injury activate skin DCs by engaging innate receptors.^5^ After their activation, DCs migrate from the skin graft to the recipient’s draining lymph nodes, delivering the required signals to activate alloreactive T cells.^3^ Murine skin conventional DCs (cDCs) can be classified into 2 main subsets with distinct functions and developmental requirements. On one hand, the cDC1 lineage is specialized for cross-presenting antigens to CD8^+^ T cells.^6–8^ cDC1s express CD103 and XCR1 and depend on the transcription factors Batf3 and Irf8 to develop.^9,10^ On the other hand, cDC2 development requires ZEB-2 and E2–2.^11,12^ cDC2s express CD11b and CD172a (SIRPα) and better stimulate CD4^+^ T cells than cDC1s.^13–15^ Based on transcriptional profiles at the single-cell level, cDC2s are proposed to be a heterogeneous group comprising the distinct subsets DC2 and DC3.^16^ Whether DC2 and DC3 represent distinct lineages or different stages of maturation remains to be formally established. Moreover, cDCs have developmental origins, surface markers, and functions that are distinct from those of monocyte-derived DCs (Mo-DCs).^17,18^ In humans, CD141^+^ and CD1c^+^ are the functional counterparts of murine cDC1s and cDC2s, respectively.^3^ Murine cDC1s have been demonstrated to have an important role in initiating the rejection of major^19^ and minor^20^ allogeneic antigens in skin transplantation. However, the specific contribution of cDC subsets to the rejection of human VCAs remains unknown.

In this study, we evaluated human cDC subsets and their maturation status at rejection and nonrejection time points in patients who underwent upper extremity and face transplantation. We also compared the DC maturation status of skin allografts to that of native skin tissue from the same subject. Finally, using a humanized skin transplant model, we demonstrated that the local modulation of cDC1s decreased alloreactive T cells and increased regulatory T cells in the target tissue. Therefore, cDC1s play an important role in rejecting human skin allografts and may thus represent a novel therapeutic target for the modulation and fate of alloimmune responses.

## 2. Materials and methods

### 2.1. Vascular composite allograft transplant subjects

The study included 6 patients who received facial transplants and 3 who received upper extremity transplants at the Brigham and Women’s Hospital. Before participation, patients were evaluated by our multidisciplinary VCA transplant team. Donors and recipients were matched according to sex, skin color, and ABO compatibility in addition to a negative T and B cell cytotoxic crossmatch. Demographic details are displayed in the Table. All patients provided written informed consent to participate in the clinical trial ClinicalTrials.gov NCT01281267 for facial transplantation and NCT01293214 for upper extremity transplantation, as approved by the Human Research Committee at the Mass General Brigham (MGB IRB 2009P001719).

### 2.2. Patient immunosuppression

All patients received mycophenolate mofetil (1000 mg), methylprednisolone (500 mg), and rabbit anti-thymocyte globulin (1.5 mg/kg/d for 4 days) for induction therapy starting at the time of transplantation. Maintenance immunosuppression consisted of mycophenolate mofetil (initially 1000 mg twice daily and reduced to 500–750 mg twice daily for the long term), tacrolimus (adjusted to achieve target levels of 10–15 ng/mL in the first 6 months, followed by 8–12 ng/mL for up to 1 year and 6–10 ng/mL thereafter), and prednisone taper (down to 20 mg on day 5 and 5–7.5 mg for the long term). Prednisone withdrawal was attempted in patient 1, but due to the higher occurrence of acute rejection episodes during winter months, low-dose prednisone (5 mg) was seasonally reinitiated. Perioperative antibacterial prophylaxis consisted of vancomycin and cefazolin and was modified according to perioperative findings. All patients received trimethoprim-sulfamethoxazole and valganciclovir prophylaxis against *Pneumocystis jirovecii* and cytomegalovirus, respectively, for ≥6 months. In the presence of clinical acute cellular rejection, patients were treated with pulse solumedrol 500 mg/d for 3 days, followed by a taper. Acute rejection episodes with no clinical signs were treated with increased maintenance immunosuppression and closely followed. Topical steroids or tacrolimus were also used as adjuvant therapy. In case of no response, further T cell–depletion therapy (rabbit anti-thymocyte globulin, alemtuzumab) was attempted. Additional methodological details are found online in the Supporting Information section.

### 2.3. Statistics

We used a Wilcoxon test for paired 2-group comparisons. We used an unpaired Student’s t-test in animal experiments to compare the 2 independent groups. All statistical tests were 2-sided with a type 1 error rate of 0.05 to determine statistical significance. Prism software was used for data analysis and graph drawing (GraphPad Software, Inc).

## 3. Results

### 3.1. Circulating and skin cDC1s are expanded during VCA rejection

**Figure 1.**
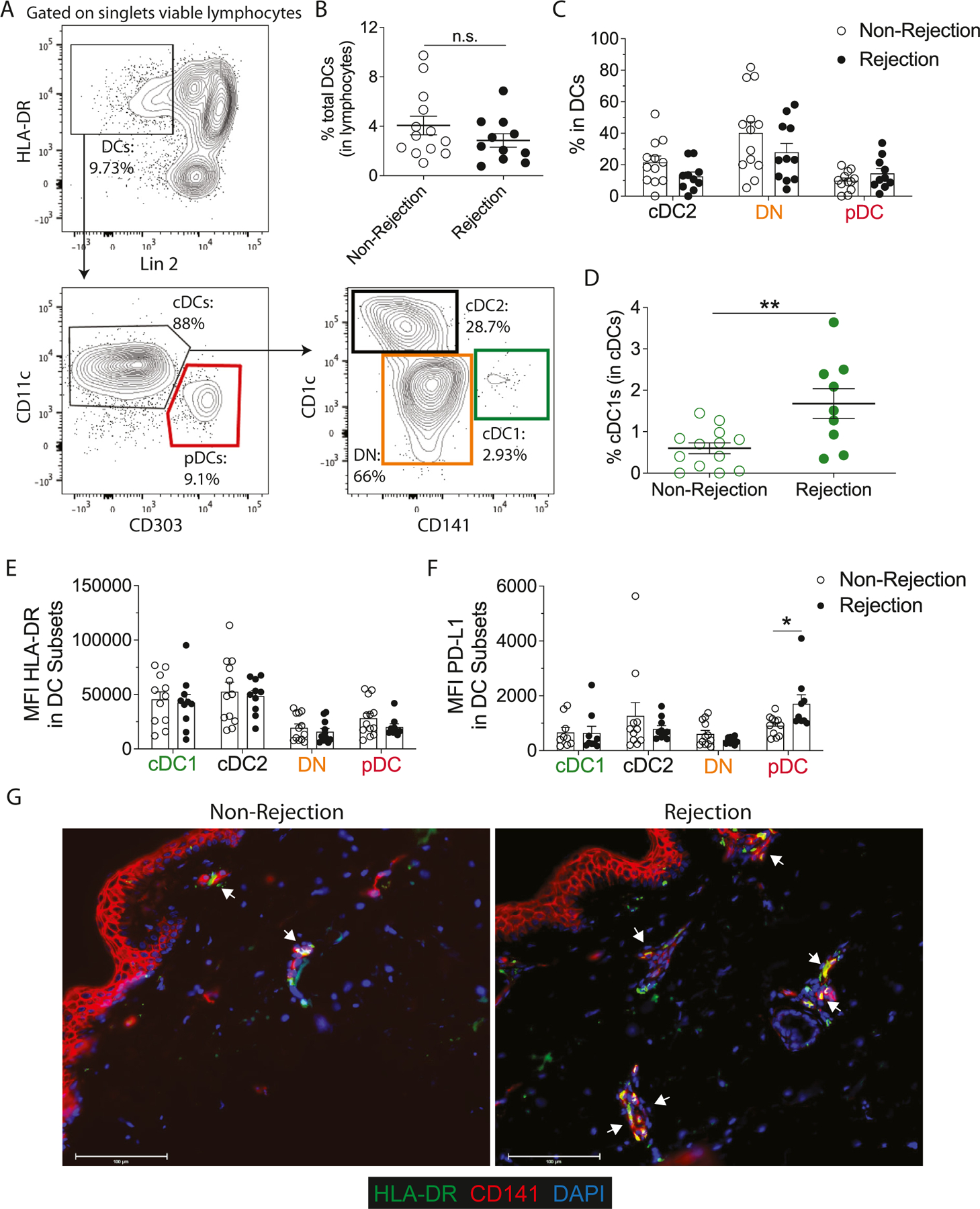
(A) Gating strategy used for the characterization of circulating DC subsets: plasmacytoid DCs (pDCs: Lin2^−^HLA-DR^+^CD11c^low/−^CD303^+^), conventional DCs type I (cDC1s: Lin2^−^HLA-DR^+^CD11c^+^CD141^+^CD1c^−^), conventional DCs type II (cDC2s: Lin2^−^HLA-DR^+^CD11c^+^CD141^low/−^D1c^+^), double-negative conventional DCs (DN: Lin2^−^HLA-DR^+^CD11c^+^CD141^−^CD1c^−^). Lin2: Lineage 2 (CD3, CD14, CD19, CD20, CD56). Percentages of (B) total DCs, (C) DC subsets, and (D) cDC1s from peripheral blood at rejection and nonrejection time points. Expression of (E) HLA-DR and (F) PD-L1 DC subsets from peripheral blood at rejection and non-rejection time points. (B-F) Statistical analysis was performed using a Wilcoxon test. Data from 12 time points pooled from 9 vascularized composite allotransplantation patients and represented as mean ± SD. Some stable time points and rejection episodes are from the same patient. n.s., not significant; *P <.05; **P <.01. (G) Representative immunofluorescence of cDC1s (HLA-DR^+^CD141^+^) from the allograft of a face transplant recipient at nonrejection and rejection time points, 200× (scale bars = 100 μm). DAPI, 4’,6-diamidino-2-phenylindole; DC, dendritic cell; HLA, human leukocyte antigen; MFI, mean fluorescence intensity; PD-L1, programmed cell death ligand 1.

We recently demonstrated that the ablation of cDC1s can prolong allograft survival in a mouse model of major mismatch skin transplantation.^19^ Based on this finding, we reasoned that human cDC1s could be involved in human skin rejection in VCA. We characterized DC subsets from peripheral blood (the gating strategy is shown in Fig. 1A) and skin biopsies of 9 individuals who underwent upper extremity or face transplantation in our institution. The clinical characteristics of these individuals are detailed in the Table. We observed no significant changes in the percentages of circulating total DCs between nonrejection and rejection time points (Fig. 1B). However, a detailed subset analysis revealed a significant increase in circulating cDC1s (CD141^+^) during rejection compared to nonrejection time points (Fig. 1C, D). In contrast, no significant changes were observed in the frequencies of cDC2s (CD1c^+^), plasmacytoid DCs (pDCs) (CD303^+^), or DN (CD141^−^CD1c^−^) DCs (Fig. 1C). To assess the maturation status of DCs, we evaluated the expression of human leukocyte antigen (HLA)-DR and programmed cell death ligand 1 (PD-L1) across DC subsets. While no changes were observed in the HLA-DR levels of circulating DCs (Fig. 1E), PD-L1 was upregulated in pDCs during rejection (Fig. 1F). Since the DCs could be modulated in the tissue during rejection, we evaluated cDC1s in skin biopsies from our VCA patients by immunofluorescence. cDC1s are one of the main tissue-resident and migratory subsets of human skin and are defined as CD141^+^HLA-DR^+^ cells.^21^ We found a higher number of skin cDC1s at rejection than at nonrejection time points (Fig. 1G). Thus, circulating and tissue CD141^+^ cDC1s are upregulated during VCA rejection.

### 3.2. Increased presence and enhanced maturation of cDC1s in nonrejecting allografts compared to native skin

**Figure 2.**
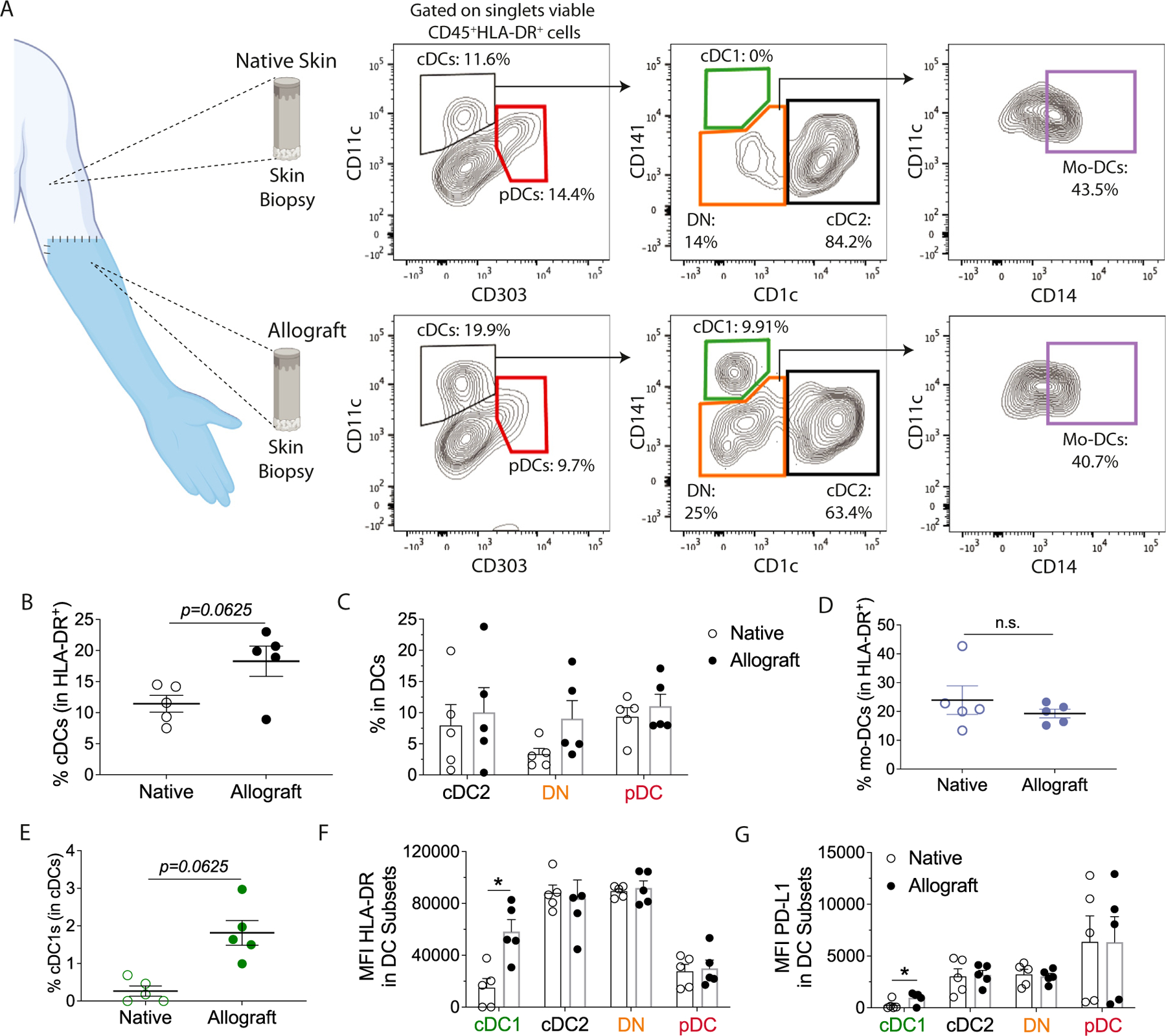
DC profiling in the allograft and native skin of vascularized composite allotransplantation (upper extremities) recipients. (A) Gating strategy used to characterize of skin-infiltrating DC subsets: plasmacytoid DCs (pDCs: CD45^+^HLA-DR^+^CD11c^low/−^CD303^+^), conventional DCs type I (cDC1s: CD45^+^HLA-DR^+^CD11c^+^CD141^+^CD1c^−^), conventional DCs type II (cDC2s: CD45^+^HLA-DR^+^CD11c^+^CD141^low/−^CD1c^+^), double-negative conventional DCs (DN: CD45^+^HLA-DR^+^CD11c^+^CD141^−^CD1c^−^), monocyte-derived DCs (Mo-DCs: CD45^+^HLA-DR^+^CD11c^+^CD141^−^CD1c^−^CD14^+^). Proportions of skin-infiltrating (B) total cDCs, (C) cDC2s, DN, and pDC subsets, and (D) Mo-DCs. (E) Percentages of skin-infiltrating cDC1s. Expression of (F) HLA-DR and (G) PD-L1 by skin-infiltrating DC subsets. (B-G) Statistical analysis was performed using a Wilcoxon test. Data from 5 debulking surgeries pooled from 3 patients and represented as mean ± SD. DC, dendritic cell; HLA, human leukocyte antigen; MFI, mean fluorescence intensity; n.s., not significant; PD-L1, programmed cell death ligand 1.

In VCA, the small sample sizes obtained from punch biopsies technically limit our ability to characterize and compare DCs infiltrating human allografts to native tissues. Therefore, we utilized debulking surgeries on upper extremity transplant recipients to analyze DC subsets and their maturation status within the graft microenvironment during nonrejection (Fig. 2A). We compared these findings to those of the recipient’s adjacent native skin removed during the procedure. Allograft and native skin samples were processed, and the infiltrating cells were isolated and stained for flow cytometric analysis. We observed increased total cDCs in allografts than in native skin (Fig. 2B). Immune phenotyping of specific DC subsets demonstrated no significant differences in cDC2s, pDCs, DN DCs (Fig. 2C), or Mo-DCs (Fig. 2D), while extremity allografts had higher frequencies of cDC1s than native skin (Fig. 2E). Regarding their maturation status, we observed higher levels of HLA-DR (Fig. 2F) and PD-L1 (Fig. 2G) in the cDC1s from the allografts. HLA-DR and PD-L1 levels were similar in cDC2s, pDCs, and DN DCs from the allograft and native skin (Fig. 2F, G). To evaluate a potential role of DC3 in rejection, we gated CD14^+^ cells inside the cDC2 population (Supplementary Fig. 1A) and found no differences between extremity allografts and native skin tissue (Supplementary Fig. 1B, C). Our data suggest that the graft tissue with alloantigens favors the accumulation and activation of cDC1s in the VCA graft microenvironment.

### 3.3. Local modulation of cDC1s decreased skin alloimmunity

**Figure 3.**
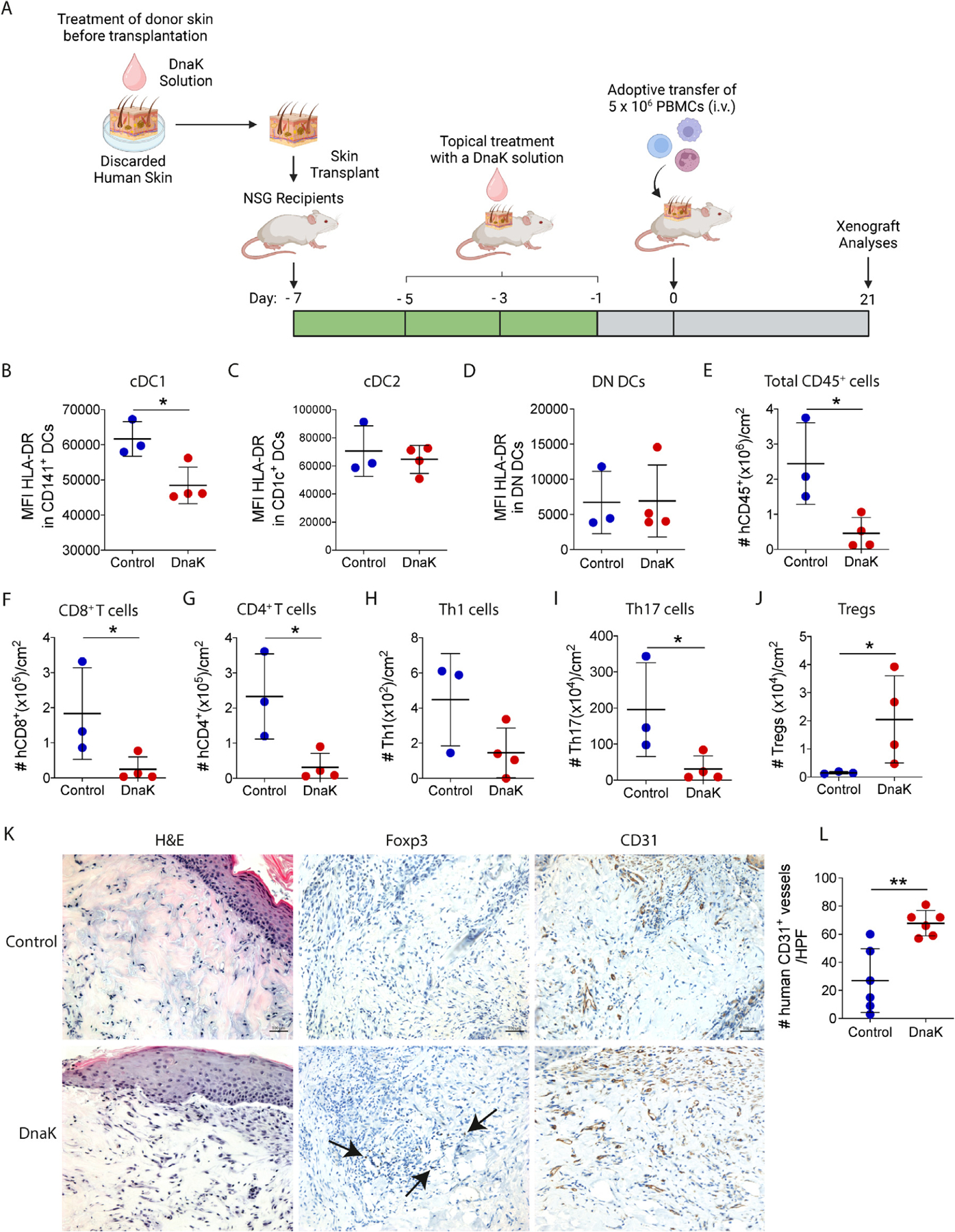
Modulation of skin cDC1s by mycobacterial DnaK leads to local immune regulation and decreased allograft injury. (A) Discarded human skin was embedded in a solution with mycobacterial DnaK or vehicle (1× phosphate-buffered saline, control group) for 2 hours and transplanted onto NSG recipient mice. To locally target DCs, skin xenografts were topically treated with DnaK or vehicle every 2 days. Seven days posttransplant, recipients were injected with 5 × 10^6^ allogeneic PBMCs. Skin xenografts were analyzed by histology and flow cytometry on day 21 posttransplant. Expression of HLA-DR by skin-infiltrating (B) CD141^+^ cDC1s, (C) CD1c^+^ cDC2s, or (D) DN DCs. Absolute numbers of skin-infiltrating (E) total human CD45^+^ cells, (F) total CD8^+^ T cells, (G) total CD4^+^ T cells, (H) Th1 cells, (I) Th17 cells, and (J) Tregs in DnaK-treated or control groups (n = 3–4 mice per group). See also Supplementary Figure S2. (B-J) All data were normalized by cm^2^ of tissue. (K) Representative images of H&E staining and immunohistochemical stainings for human Foxp3 and CD31, 200× (scale bars = 100 μm). (L) Absolute counts of human CD31^+^ vessels in the skin xenografts. The number of positive cells was quantified from 2 representative 400× fields from 3 transplanted xenografts. Statistical analysis was performed using a t-test. cDC, conventional dendritic cell; DC, dendritic cell; DN, dominant-negative; i.v., intravenous; H&E, hematoxylin and eosin; HLA, human leukocyte antigen; MFI, mean fluorescence intensity; PBMC, peripheral blood mononuclear cell; Th, T helper cell; Treg, regulatory T cell.

Our group previously demonstrated that topical treatment with the mycobacterial protein DnaK modulates murine skin allograft DCs, decreasing donor Major Histocompatibility Complex class II (MHCII) and significantly improving skin allograft survival in mice.^19^ Using a humanized skin transplant model, we investigated whether the local immune modulation of cDC1s could decrease human skin immunogenicity and reduce graft injury. In this model, we transplanted human skin onto genetically immunosuppressed NOD.Cg-PrkdcscidIl2rgtm1Wjl/SzJ (NSG) mice. Before transplantation, human skin grafts were immersed in a solution of 1× phosphate-buffered saline alone or containing mycobacterial DnaK for 2 hours on ice. Skin xenografts were transplanted and topically treated with DnaK solution or control every 48 hours for 7 days. We then administered allogeneic peripheral blood mononuclear cells intravenously 1 week after the skin transplant (Fig. 3A). Skin xenografts were evaluated for immune cell content and tissue injury 21 days after the transfer of the peripheral blood mononuclear cells (gating strategy in Supplementary Fig. 2). Topical treatment with DnaK significantly decreased HLA-DR expression in CD141^+^ cDC1s (Fig. 3B) but had no effect on CD1c^+^ cDC2s (Fig. 3C) or DN DCs (Fig. 3D). DnaK treatment did not alter the absolute numbers of cDC1s, cDC2s, or DN DCs within the graft (Supplementary Fig. 3).

Importantly, DnaK-mediated DC modulation decreased recruitment of allogeneic CD45^+^ leukocytes in the skin graft (Fig. 3E). Given that DCs are the main activators of T cells, we next evaluated T cell recruitment to the xenografts. DnaK-treated grafts showed significantly fewer CD8^+^ T cells (Fig. 3F) and CD4^+^ T cells (Fig. 3G) per skin cross-sectional area than controls. Furthermore, T helper 1 (Th1) cells were markedly reduced (Fig. 3H), and Th17 cells were significantly decreased (Fig. 3I), indicating suppression of proinflammatory T cell responses. Concomitantly, we observed enhanced numbers of regulatory T cells (Tregs) in skin allografts treated with DnaK (Fig. 3J), consistent with DnaK immune regulatory effects.^22^ Histologically, xenografts treated with DnaK showed increased Foxp3^+^ Tregs, which correlated with less vascular endothelium injury (Fig. 3K) demonstrated through increased CD31^+^ staining compared to controls (Fig. 3L). Altogether, our data suggest that DnaK treatment can specifically modulate human cDC1s and reshape the immune landscape within the skin microenvironment, leading to decreased tissue injury and enhanced immune regulation.

## 4. Discussion

This study investigated different DC subsets in the peripheral blood and skin of VCA recipients. Our data indicate that circulating and graft tissue CD141^+^ cDC1s, but not other DC subsets, were elevated during rejection time points. Additionally, we demonstrated that cDC1s were more abundant and exhibited increased maturation in human VCA grafts compared to native skin from the same individual. Using a humanized skin transplant model, we found that local treatment of human skin grafts with mycobacterial DnaK selectively modulates tissue cDC1s and alters the profile of immune cells infiltrating the skin microenvironment, resulting in reduced tissue injury.

Innate immunity is crucial for initiating and dictating adaptive immune responses against transplanted organs.^23,24^ DCs are the most effective APCs among innate immune cells. DCs become activated after transplantation, prompting their migration to the host’s secondary lymphoid tissues and the engrafted tissue,^25,26^ activating T cells. Clinical data from kidney transplantation corroborate these preclinical observations: a high density of graft-infiltrating DCs is associated with increased T cell infiltration, diminished allograft function, and reduced survival in kidney transplant recipients.^27^ However, the precise role of different DC subsets in VCA requires further elucidation.

The skin is an immunologically active tissue with a complex network of immune cells, including an extensive network of T cells^28^ and a dynamic population of APCs.^3^ The skin is historically categorized as the most immunogenic organ.^29^ We used debulking surgeries to perform DC profiling in the allograft microenvironment and compared it to the adjacent native skin of the same individual. The allograft tissue demonstrated a higher proportion of cDC1s with an activated phenotype. Comparing the allograft immune landscape with adjacent native tissue is essential, as the skin from different body regions exhibits considerable differences in immunological composition.^30^ In addition, exposure to various external physical and chemical insults in different locations influences the skin microbiome and the local immune response, leading to generic inflammation that may mimic alloimmune injury.^31,32^ Given that the allograft and the nearby native skin were exposed to identical external factors, our findings indicate a more immunologically active environment in the allograft. This heightened activity is likely due to the persistent local release of alloantigens, which primarily stimulate adaptive immunity and possibly innate memory alloreactive responses.^23^ This increased DC activity could lead to a higher presence of T cells infiltrating the VCA allografts than native skin, as previously demonstrated by our group.^33^

In mice, the cDC1 subset expresses CD103 and XCR1, requiring the transcription factors Batf3 and Irf8 for their development. cDC1s are specialized in priming CD8^+^ T cells via cross-presentation.^34^ Historically, skin cDC1s were found to cross-present skin pathogen-associated antigens and elicit CD8 T cell responses.^6,35^ Moreover, this subset contributes to autoimmune skin disorders by shaping the activation of autoreactive T cells, perpetuating the cycle of inflammation and tissue damage.^36^ The importance of cDC1s in transplant rejection is underscored by the observation that skin grafts lacking cDC1s (from *Batf3*^−*/*−^ donors) exhibit improved survival, along with reduced expression of donor MHCII in the draining lymph nodes and allografts, as well as fewer primed T cells.^19^ Additionally, cDC1s are key amplifiers of graft-versus-host disease pathology^37^ and also have been implicated in the rejection of minor mismatched grafts.^20^ Here, we extended the relevance of cDC1s in human face and extremity transplantation. Thus, cDC1s are critical in transplant rejection and skin autoimmune disorders, highlighting their potential as key targets for future therapeutic interventions. However, cDC1s are also reported to be involved in the generation and homeostasis of Tregs, especially in noninflammatory conditions.^38,39^ This suggests that their function may be highly context-dependent, shifting between pro-rejection and tolerogenic roles depending on local immune cues in the tissue microenvironment. Given the inflammatory status of Mo-DCs^40^ and DC3,^41^ we unexpectedly observed no differences between these subsets and the analyzed groups, highlighting the skin’s unique immunological features.

Immunosuppressive regimens used in VCA clinical practice primarily target adaptive immune cells and have many systemic side effects.^1^ Therefore, developing novel therapies targeting innate immunity is a promising strategy to overcome this problem. Thus, understanding the DC subsets and their activation in vivo may enable effective immune modulation with lower doses of immunosuppressants, thereby minimizing drug toxicity and side effects. One of the advantages of VCAs over solid organ transplantation is the potential to apply topical treatments that may minimize systemic toxicity. In this context, the mycobacterial protein DnaK represents a promising candidate. We and others have shown that extracellular mycobacterial DnaK primarily exerts its immunomodulatory effects indirectly, through binding to innate receptors such as Siglec-E and LOX-1 on DCs^42^—particularly cDC1s^19^—leading to their downregulation and the induction of regulatory T cells.^43,44^ Importantly, DnaK does not directly suppress T cells; rather, it modulates the antigen-presenting environment that shapes T cell responses.^43,45^ Indeed, in our model, local pretreatment of human skin grafts with mycobacterial DnaK resulted in reduced expression of HLA-DR, increased Tregs, and decreased infiltration of CD4^+^ and CD8^+^ effector T cells. In line with these findings, Ashraf et al^4^ recently demonstrated in an experimental model of hind limb transplantation that the depletion of donor DCs led to reduced graft injury, which correlated with attenuated CD8 and Th17-mediated immune responses. These data underscore the role of skin DCs not only in initiating systemic T cell responses via lymphoid priming but also in activating skin-resident memory T cells locally.^46^ We propose that mycobacterial DnaK modulates the local immune environment in VCA by targeting cDC1s, thereby limiting inflammation and alloimmune infiltration. These findings highlight skin cDC1s as a key therapeutic target for locally modulating VCA rejection, offering a potential strategy for reducing systemic immunosuppression and its associated toxicity.

The limitations of this study include the small number of patients evaluated and the need to validate our findings in other VCA cohorts from different centers. Additionally, while we examined cDC2s as a group, we did not formally differentiate between DC2 and DC3 subsets, which may have distinct immunological roles. While the humanized skin transplant model does not fully replicate the complexity of the human immune system, it effectively models clinically relevant features of VCA rejection, in which the epidermis is the primary site of immune-mediated injury. In clinical practice, epidermal infiltration by T cells defines the severity of rejection according to Banff criteria.^47^ Consistent with this, we assessed rejection severity in our model through histological evaluation (hematoxylin and eosin staining) and CD31 immunostaining to reflect vascular integrity and graft injury.^48^ Thus, despite inherent limitations, this model provides a valuable platform to dissect the mechanisms of human skin allograft rejection and evaluate localized immune-modulating interventions.

Overall, our findings underscore the critical role of CD141^+^ cDC1s in VCA rejection, highlighting their potential as therapeutic targets. By demonstrating the effectiveness of localized treatments, such as the mycobacterial DnaK protein, in modulating the immune environment and reducing tissue injury, this research opens new avenues for minimizing systemic toxicity in VCA through targeted, localized immunosuppressive strategies. These insights provide background for developing more precise and less harmful approaches to managing VCA rejection, ultimately improving patient outcomes.

## Supplementary Material

1

## Figures and Tables

**Table:** Baseline characteristics of vascularized composite allograft recipients and donors.

Characteristics	Patient 1	Patient 2	Patient 3	Patient 4	Patient 5	Patient 6	Patient 7	Patient 8	Patient 9
**Recipient**									
Age at transplant, y	57	25	30	44	38	33	65	40	30
Sex	Female	Male	Male	Female	Male	Male	Male	Male	Male
Ethnicity	White	White	White	White	White	White	White	White	White
Cause of injury	Animal attack	Electrical burn	Electrical burn	Chemical burn	Gunshot	Gunshot	Septic shock	Septic shock	Ballistic trauma
Surgery	Face, bilateral upper extremity	Face	Face	Face	Face	Face	Bilateral forearm	Bilateral upper extremity	Bilateral upper extremity
PRA (%)	0	68	0	97	22	32	0	69	0
Donor-specific antibodies	Negative	Negative	Negative	Positive	Negative	Positive	Negative	Positive	Negative
HLA mismatch (A, B, DR)	8	8	5	11	8	7	5/6	5/6	4/6
CMV status	Positive	Positive	Negative	Positive	Negative	Positive	Negative	Negative	Negative
EBV status	Positive	Positive	Positive	Positive	Positive	Positive	Positive	Positive	Positive
Induction agent	Thymoglobulin	Thymoglobulin	Thymoglobulin	Thymoglobulin	Thymoglobulin	Thymoglobulin	Thymoglobulin	Thymoglobulin	Thymoglobulin
**Donor**									
Age, y	42	48	31	56	51	23	44	23	27
Sex	Female	Male	Male	Female	Male	Male	Male	Male	Male
CMV status	Positive	Positive	Positive	Negative	Positive	Negative	Negative	Negative	Negative
EBV status	Positive	Positive	Positive	Positive	Positive	Positive	Positive	Positive	Positive
Total ischemia time, h	2	4	2	3	3	1.5	4	4	4 right/5 left

CMV, cytomegalovirus; EBV, Epstein-Barr virus; HLA, human leukocyte antigen; PRA, panel reactive antibody.

## Data Availability

All relevant data in the article and its supplementary information files can be accessed on request.

## Appendix A. Supplementary data

Supplementary data to this article can be found online at https://doi.org/10.1016/j.ajt.2025.04.016.

## Abbreviations:

APC: antigen-presenting cell
cDC: conventional dendritic cell
DC: dendritic cell
DN: double-negative
HLA: human leukocyte antigen
Mo-DCs: monocyte-derived dendritic cell
pDC: plasmacytoid dendritic cell
PD-L1: programmed cell death ligand 1
Th: T helper cell
Treg: regulatory T cell
VCA: vascularized composite allotransplantation
