# Supplementary material for: Human type 1 conventional dendritic cells contribute to skin transplant rejection
Source: Am J Transplant. Author manuscript; Available in PMC 2026 May 19. (PMC13186244; doi:10.1016/j.ajt.2025.04.016)
Supplement: 1 [file NIHMS2174848-supplement-1.docx]

**Supporting Information**

**Human Type 1 Conventional Dendritic Cells Contribute to Skin Transplant Rejection**

*Borges et al.*

**Supporting Materials and Methods**

*2.3 Skin xenotransplant model*

Human skin tissues were obtained as discarded tissue from plastic surgery (panniculectomy specimens, MGB IRB 2016P001844) and immersed in a solution of PBS 1x alone or containing 30 μg/ml of *M. tuberculosis* (mycobacterial) DnaK for 2h on ice, as previously described by our group ^1^. After that, a 1 cm^2^ section of full-thickness human skin was transplanted on the dorsum of six-to-eight weeks-old NSG recipients using a sterile monofilament, non-absorbable suture, as previously described ^2,3^. Skin xenografts were topically treated with a solution of PBS 1x alone or containing 30 μg/ml of *M. tuberculosis* DnaK every 48h for seven days. On day 7 after the transplantation, each mouse was intravenously injected with 5x10^6^ PBMCs from a different donor (allo-PBMCs). Skin xenografts were photographed daily and monitored for signs of rejection, primarily changes in color and necrosis. Skin allografts and peripheral blood were harvested for analyses on day 21 after the PBMC adoptive transfer. Recombinant *M. tuberculosis* DnaK was produced as previously described by our group ^1,4^. All animals were housed following the Institutional Animal Care and Use Committee (IACUC) and National Institutes of Health (NIH) Animal Care guidelines. The Mass General Brigham IACUC approved all experiments (protocol numbers 2016N000250).

*Isolation and quantification of skin cells*

Immune cells from debulking surgeries or skin xenografts were isolated, as described previously ^22^. After harvesting, skin tissues were recovered overnight in RPMI media (Lonza) supplemented with 20% FBS, 100 mM L-glutamine and penicillin/streptomycin at 4°C. After that, the tissues were minced into small pieces in 10% FBS-supplemented RPMI, followed by incubation in Collagenase D (Sigma, 0.2%) and DNAse (30 Kunitz Units/mL, Invitrogen) at 37 °C for 2 h with shaking (350 rpm). Cells were passed through a 70 µm cell strainer, washed and recovered in RPMI media (Lonza) supplemented with 20% FBS, 100 mM L-glutamine and penicillin/streptomycin for 4h at 37 °C. We quantified the total skin cell numbers using fluorescent AccuCheck Counting Beads (Invitrogen) by flow cytometry. Each skin sample had its area calculated, and all data were normalized by skin area (in cm^2^).

*Flow cytometry*

We stained PBMCs, skin cells from debulking surgeries, and xenografts for flow cytometry. PBMCs from different time points were thawed, washed and stained on the same day to avoid variability. Anti-human antibodies directed against CD45 (clone HI30, 1:100), CD303 (clone 201A, 1:20), CD11c (clone 3.9, 1:20), HLA-DR (clone L243, 1:20), CD141 (clone M80, 1:20), CD1c (clone L161, 1:20), PD-L1 (clone MIH3, 1:20), CD3 (clone OKT3, 1:100), CD19 (clone HIB19, 1:100), and CD14 (M5E2, 1:50) were from Biolegend; lineage cocktail 2-FITC (CD3, CD14, CD19, CD20, CD56, 1:10) was from BD Biosciences; and CD14 (clone RMO52, 1:50) was from Beckman Coulter. Thawed PBMCs and recovered skin cells were Fc-blocked (Miltenyi) for 20 min before staining for surface markers for 30 minutes in FACS buffer (2% FBS in PBS 1x) on ice. Stained PBMCs were analyzed on a FACS Canto II flow cytometer (BD Biosciences) with FACSDiva software (BD Biosciences). Data were analyzed using FlowJo software (TreeStar). Viable cells were selected based on the staining with Fixable Viability Dye eFluor 780 (eBioscience) or Zombie NIR Fixable Viability Kit (Biolegend). The gating strategy is displayed in **Supplemental Figure 2**.

**
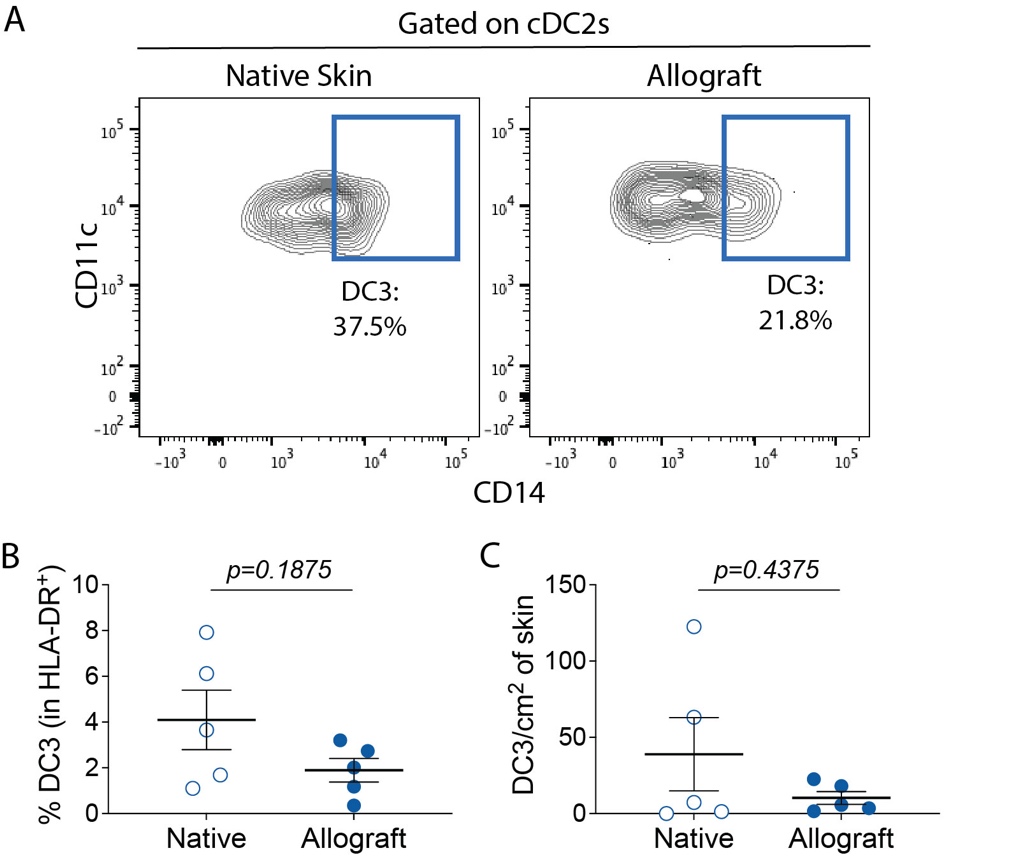
**

**Supplemental Figure 1. Skin DC3 in the allograft and native skin of VCA recipients.** All samples were analyzed with FlowJo software. Viability = Fixable viability dye. (**A**) Gating strategy used to characterize skin-infiltrating D3 subset: CD14+ cells in conventional DCs type II (cDC2s: CD45^+^HLA-DR^+^CD11c^+^CD141^low/-^CD1c^+^). (**B**) Percentages and (**C**) absolute numbers of skin DC3 in native skin and limb allografts. Statistical analysis was performed using a Wilcoxon test. Data from five debulking surgeries pooled from three patients and represented as mean ± SD.


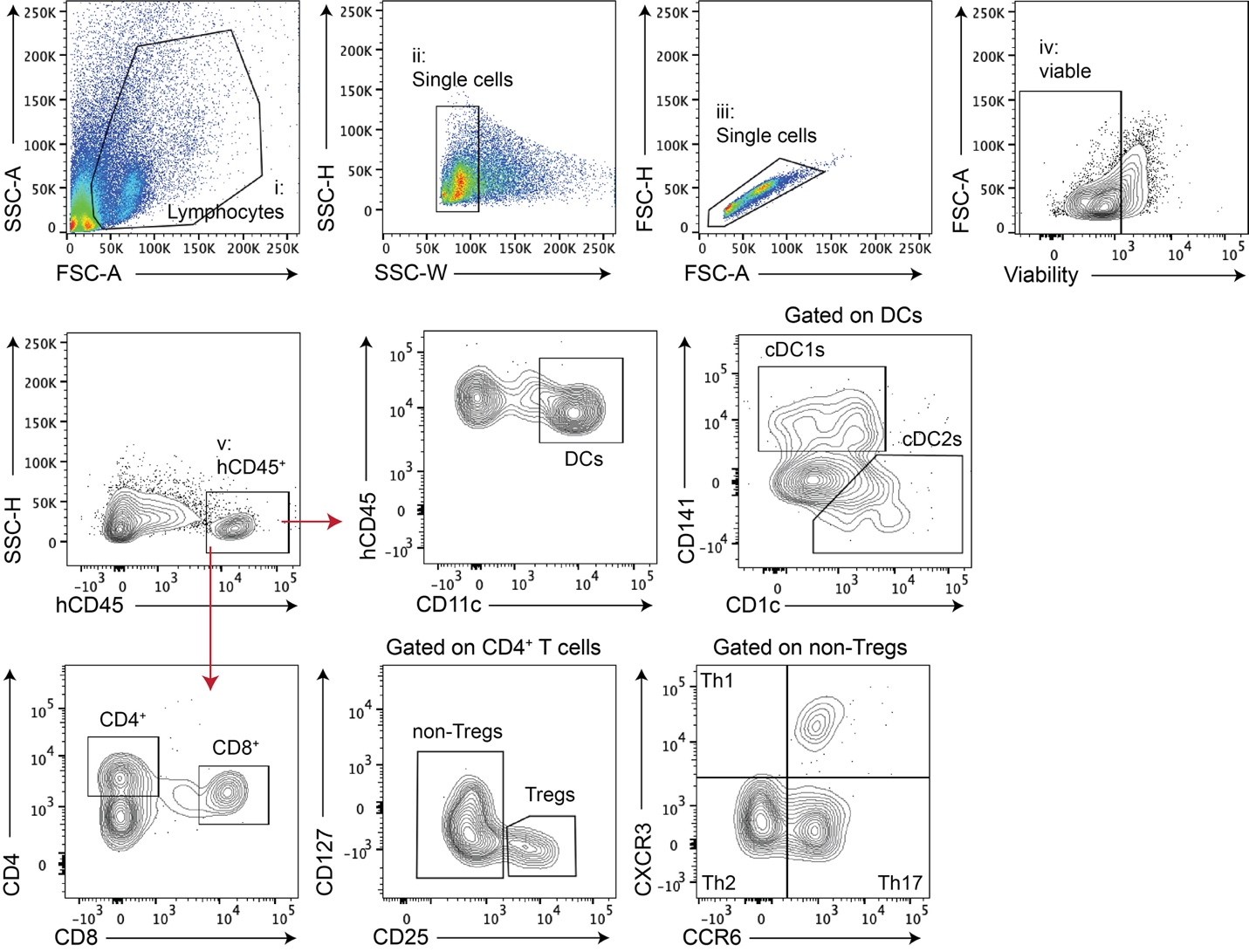


**Supplemental Figure 2. The gating strategy of the skin-infiltrating immune cell populations analyzed in Figure 3.** All samples were analyzed with FlowJo software. Viability = Fixable viability dye.

**
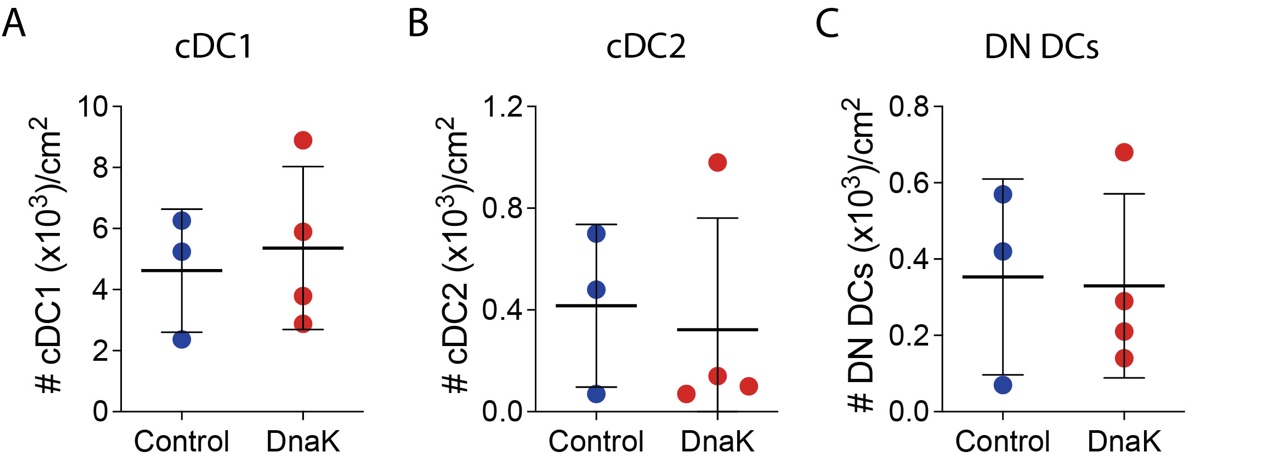
**

**Supplemental Figure 3. DnaK treatment does not change the number of skin DC subsets.** Numbers of skin-infiltrating (**A**) CD141^+^ cDC1s, (**B**) CD1c^+^ cDC2s, (**C**) or DN DCs in DnaK-treated or control groups (n = 3 - 4 mice per group) from **Figure 3**. All data were normalized by cm^2^ of tissue.

**Supplemental References**

1. Borges TJ, Murakami N, Machado FD, et al. March1-dependent modulation of donor MHC II on CD103+ dendritic cells mitigates alloimmunity. *Nature Communications*. 2018 2018;9(1):3482. doi:10.1038/s41467-018-05572-z

2. Magee CN, Murakami N, Borges TJ, et al. Notch-1 Inhibition Promotes Immune Regulation in Transplantation Via Regulatory T Cell–Dependent Mechanisms. *Circulation*. 2019 2019;140(10):846-863. doi:10.1161/circulationaha.119.040563

3. Borges TJ, Abarzua P, Gassen RB, et al. T cell-attracting CCL18 chemokine is a dominant rejection signal during limb transplantation. *Cell Reports Medicine*. 2022/03/15/ 2022;3(3)doi:10.1016/j.xcrm.2022.100559

4. Lopes RL, Borges TJ, Zanin RF, Bonorino C. IL-10 is required for polarization of macrophages to M2-like phenotype by mycobacterial DnaK (heat shock protein 70). *Cytokine*. 2016 2016;85:123-129. doi:10.1016/j.cyto.2016.06.018
